# Does the Degree of Trunk Bending Predict Patient Disability, Motor Impairment, Falls, and Back Pain in Parkinson's Disease?

**DOI:** 10.3389/fneur.2020.00207

**Published:** 2020-03-31

**Authors:** Christian Geroin, Carlo Alberto Artusi, Marialuisa Gandolfi, Elisabetta Zanolin, Roberto Ceravolo, Marianna Capecci, Elisa Andrenelli, Maria Gabriella Ceravolo, Laura Bonanni, Marco Onofrj, Roberta Telese, Giulia Bellavita, Mauro Catalan, Paolo Manganotti, Sonia Mazzucchi, Sara Giannoni, Laura Vacca, Fabrizio Stocchi, Miriam Casali, Cristian Falup-Pecurariu, Maurizio Zibetti, Alfonso Fasano, Leonardo Lopiano, Michele Tinazzi

**Affiliations:** ^1^Neurology Unit, Movement Disorders Division, Department of Neurosciences, Biomedicine and Movement Sciences, University of Verona, Verona, Italy; ^2^Department of Neuroscience “Rita Levi Montalcini”, University of Torino, Torino, Italy; ^3^Neuromotor and Cognitive Rehabilitation Research Center, Department of Neurosciences, Biomedicine and Movement Sciences, University of Verona, Verona, Italy; ^4^Neurorehabilitation Unit, Azienda Ospedaliera Universitaria Integrata, Verona, Italy; ^5^Department of Public Health and Community Medicine, University and Hospital Trust of Verona, Verona, Italy; ^6^Department of Clinical and Experimental Medicine, University of Pisa, Pisa, Italy; ^7^Department of Experimental and Clinical Medicine, Neurorehabilitation Clinic, “Politecnica delle Marche” University, Ancona, Italy; ^8^Department of Neuroscience, Imaging and Clinical Sciences, University G.d'Annunzio of Chieti-Pescara, Chieti, Italy; ^9^Clinical Neurology Unit, Department of Medical, Surgical and Health Services, University of Trieste, Trieste, Italy; ^10^University and Institute for Research and Medical Care IRCCS San Raffaele, Rome, Italy; ^11^Department of Neurology, Faculty of Medicine, Transilvania University, Brasov, Romania; ^12^Edmond J. Safra Program in Parkinson's Disease and the Morton and Gloria Shulman Movement Disorders Clinic, Division of Neurology, Toronto Western Hospital, UHN, University of Toronto, Toronto, ON, Canada; ^13^Krembil Brain Institute, Toronto, ON, Canada

**Keywords:** Parkinson's disease, camptocormia, Pisa syndrome, anterocollis, postural abnormalities

## Abstract

**Background:** Postural abnormalities in Parkinson's disease (PD) form a spectrum of functional trunk misalignment, ranging from a “typical” parkinsonian stooped posture to progressively greater degrees of spine deviation.

**Objective:** To analyze the association between degree of postural abnormalities and disability and to determine cut-off values of trunk bending associated with limitations in activities of daily living (ADLs), motor impairment, falls, and back pain.

**Methods:** The study population was 283 PD patients with ≥5° of forward trunk bending (FTB), lateral trunk bending (LTB) or forward neck bending (FNB). The degrees were calculated using a wall goniometer (WG) and software-based measurements (SBM). Logistic regression models were used to identify the degree of bending associated with moderate/severe limitation in ADLs (Movement Disorders Society Unified PD Rating Scale [MDS-UPDRS] part II ≥17), moderate/severe motor impairment (MDS-UPDRS part III ≥33), history of falls (≥1), and moderate/severe back pain intensity (numeric rating scale ≥4). The optimal cut-off was identified using receiver operating characteristic (ROC) curves.

**Results:** We found significant associations between modified Hoehn & Yahr stage, disease duration, sex, and limitation in ADLs, motor impairment, back pain intensity, and history of falls. Degree of trunk bending was associated only with motor impairment in LTB (odds ratio [OR] 1.12; 95% confidence interval [CI], 1.03–1.22). ROC curves showed that patients with LTB of 10.5° (SBM, AUC 0.626) may have moderate/severe motor impairment.

**Conclusions:** The severity of trunk misalignment does not fully explain limitation in ADLs, motor impairment, falls, and back pain. Multiple factors possibly related to an aggressive PD phenotype may account for disability in PD patients with FTB, LTB, and FNB.

## Introduction

Postural abnormalities are common features of Parkinson's disease (PD) and manifest in over 20% of patients during the course of disease (1). Three main types of PD-associated postural abnormalities are distinguished: camptocormia (CC), defined as an involuntary forward trunk bending (FTB) appearing during standing or walking and resolving in a supine position of at least 30° at the lumbar fulcrum and/or at least 45° at the thoracic fulcrum (2); Pisa syndrome (PS), defined as a sustained lateral trunk bending (LTB) of at least 10° worsened by prolonged sitting or walking (3); and anterocollis (AC), defined as an involuntary forward neck bending (FNB) of at least 45° (3). PD studies investigating CC, PS, and AC report greater disability in patients with postural abnormalities and a higher risk of falls, back pain, and worse quality of life (1, 3–6).

However, PD-associated postural abnormalities form a spectrum of functional trunk misalignment, ranging from a “typical” parkinsonian stooped posture, with rounding of the shoulders and flexion of the hips and knees, to progressively greater degrees of trunk bending. The current criteria for the definition of CC, PS, and AC are mainly based on expert opinion (3) or expert consensus (2), leaving issues open on the classification of postural abnormalities not fulfilling the criteria for CC, PS, or AC. Given the lack of a common and comprehensive classification for postural abnormalities and the still unexplored impact of “milder” trunk flexion on disability, there is a rationale for revision of the classification of PD-associated postural abnormalities: (1) criteria for CC, PS, and AC have been decided *a priori* based on observational judgment of movement disorder experts and are not anchored to any formal measure of activities of daily living (ADLs), motor impairment, risk of falls or pain; and (2) the current cut-offs refer to very severe conditions, leaving many PD patients with potentially disabling postural abnormalities without an effective diagnosis of postural abnormalities.

In this study, our primary aim was to analyze the association between the degree of postural abnormalities and disability, expressed as limitation in ADLs, motor impairment, falls, and back pain. The secondary aim was to determine the cut-off values of trunk/neck bending that best predict the disability. We hypothesized that significant disability in patients with postural abnormalities may not be entirely related to the degrees of trunk bending and that disability may occur at lower degrees of trunk bending than the standard criteria for defining CC, PS, and AC, which identify a final stage of a complex process of trunk control impairment.

## Methods

For this study we retrieved data from a database created for a multicenter epidemiological study on postural abnormalities in PD, encompassing comprehensive information on 811 consecutive outpatients with PD attending seven tertiary movement disorder centers in Italy (1).

### Participants

The study population was patients with a diagnosis of idiopathic PD (7) presenting ≥5° of FTB, LTB or FNB as measured with a wall goniometer. Exclusion criteria were: concomitant neurological diseases known to negatively affect posture, previous major spinal surgery, skeletal, or muscle disease (i.e., vertebral fractures, spondylodiscitis, and inflammatory myopathy), and treatment with medications possibly causing posture alterations (i.e., neuroleptics and antiemetics, except for clozapine, quetiapine, and domperidone) in the 6 months prior to enrollment.

The institutional review boards of the participating centers reviewed and approved the study protocol. All patients were informed about the nature of the study and gave their written consent to participate. Authorization was obtained for disclosure (consent-to-disclose) of any recognizable persons in photographs. The study was registered at https://clinicaltrials.gov (NCT03573232).

### Procedures

At each center, patients were evaluated while on their usual drug treatment during the ON phase. Postural evaluation was performed with the use of a wall goniometer (WG) and analysis of patients' photographs during a single session by the same rater assigned before the start of the study. The following clinical and demographical features were recorded: age, gender, age at PD onset, Movement Disorders Society Unified Parkinson's Disease Rating Scale (MDS-UPDRS) part I-IV to assess PD severity (8), modified Hoehn & Yahr (H&Y) scale to assess PD stage, disease duration (in years since diagnosis), and PD phenotype (rigid-akinetic, tremor-dominant, or mixed type) (9).

### Outcome Measures

#### Evaluation of Postural Abnormalities

Two instruments were used to measure FTB, LTB, and FNB. The degree of trunk bending was calculated using the WG (2, 10, 11) and software-based measurements (SBM) with the freeware program Kinovea® (10, 12). Patients with FTB were subdivided into a lower and an upper FTB angle group according to the fulcrum level (2). For the WG measures, a rater trained at each center rated the angles independently. For SBM, an experienced rater (C.G.) was trained in the use of the freeware program Kinovea® and performed all measurements on the patients' photographs. Interobserver reliability between the two raters in the use of WG and software-based measurement to measure FTB, LTB, and FNB is good to excellent (10).

The WG was used to visually estimate the degree of bending in patients while standing or sitting (10, 11). The zero of WG was positioned at the level of the fulcrum. A line perpendicular to the ground and an imaginary line drawn from the fulcrum of bending through the C7 spinal process (or the tragus of the ear for the FNB) formed the outer angle. The minimal detectable change for the WG recorded in degrees was 5°.

**Figure 1:**
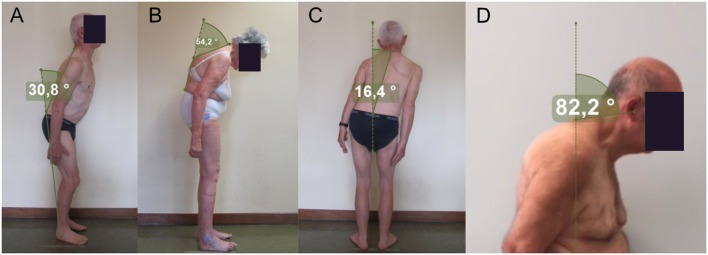
**(A)** The malleolus method; **(B)** the upper method; **(C,D)** the perpendicular method.

All patients underwent evaluation also with SBM. The lower FTB angle was calculated on the sagittal view photographs using the software-based malleolus method. A line was drawn from the L5 end of the L5/C7 line to the lateral malleolus of the foot, the external angle between these lines was measured (Figure 1A) (10, 13). The upper FTB angle was calculated on the sagittal view photographs as the outer angle between the two lines fulcrum-L5 and fulcrum-C7, with the fulcrum defined as the most distant point perpendicular to the L5/C7 line (Figure 1B) (10, 12). The LTB angle was calculated on the planar view photographs as the angle between (a) the vertical axis and (b) a line connecting the fulcrum of the bent spine with the C7 spinous process (Figure 1C) (14). The FNB was calculated on the sagittal view photographs as the angle between (a) a line connecting the C7 spinous process and the tragus of the ear, and (b) the line perpendicular to the ground (Figure 1D) (15).

#### Evaluation of Limitation in ADLs, Motor Impairment, Falls, and Pain

Limitation in ADLs was evaluated according to the MDS-UPDRS part II score (8) and motor impairment by the MDS-UPDRS part III score (8). According to the literature, an MDS-UPDRS part II score ≥17 identifies moderate/severe limitation in ADLs (16, 17) and an MDS-UPDRS part III score ≥33 moderate/severe motor impairment (18). Patients were categorized as “fallers” if they reported having sustained at least one fall in the previous month (19). A numeric rating scale (NRS) graded from 0 (no pain at all) to 10 (excruciating pain) was used to rate back pain intensity (20). A score ≥4 identified moderate/severe pain because it can interfere with the quality of life (21, 22).

### Statistical Analysis

Descriptive statistics included frequency tables and calculation of means and standard deviation (SD) for each group of patients (FTB, LTB, and FNB). In patients with combined upper and lower FTB, we used the upper fulcrum degrees as upper FTB and the lower fulcrum degrees as lower FTB.

Logistic regression models were used to estimate unadjusted and adjusted odds ratios (95% confidence interval [CI]) of MDS-UPDRS parts II, III, history of falls, NRS (dependent variables) in relation to the degree of trunk bending (independent variable); adjustment was performed taking into account the following sociodemographic and clinical features as covariates: gender, modified H&Y stage, and disease duration. Variables having clinical relevance (1) or *p* < 0.05 at univariate analysis were entered in the multivariate analysis as covariates.

When an association between the dependent variable and the degree of bending was found, sensitivity and specificity were calculated. Receiver operating characteristic (ROC) curves were constructed and the Youden index (the highest sum of values of sensitivity and specificity minus one) was calculated to obtain the optimal cut-off values of the degree of bending to identify patients with impairment. Areas under the curve (AUC) of the ROC curves were calculated to provide a measure of the overall discriminative ability of the prediction rule. Typically, a test with an AUC > 0.9 has high accuracy, while an AUC 0.7–0.9 indicates moderate accuracy, 0.5–0.7 low accuracy, and 0.5 is compared to toss-up (chance result) (23). All tests were two-tailed and considered a *p*-value < 0.05 as statistically significant. Data were analyzed using the Statistical Package for the Social Sciences (SPSS 22 for Mac, IBM-SPSS, Armonk, NY, USA).

## Results

### Clinical Features of Patients

A total of 283 PD patients were enrolled according to the inclusion and exclusion criteria. A total of 215 patients presented FTB (175 upper type, 27 lower type, and 13 both types), 88 LTB and 61 FNB. Upper FTB ranged from 21.3° to 72.3° [mean and standard deviation SBM 44.8 ± 8.6, WG 41.9° ± 8.9°]. Lower FTB ranged from 5° to 60° (SBM 28.9° ± 10.6°, WG 25.2° ± 12.4°). Coexistent upper/lower subtype ranged from 20° to 60° (SBM upper 40.7° ± 10.1°, WG 45.8° ± 11.7°; SBM lower 31.7° ± 9.2°, WG 28.1° ± 8.8°). LTB ranged from 5° to 45° (SBM 11.3° ± 7.4°, WG 11.7° ± 7.4°). FNB ranged from 15° to 106.2° (SBM 62.5° ± 20°, WG 58.7° ± 20.4°). Table 1 presents the clinical and demographical features.

**Table 1:** Clinical Features of PD Patients with one or more postural abnormality of forward trunk bending, lateral trunk bending, and forward neck bending.

**Variables**	**Patients with one or more PA**		
	**FTB**	**LTB**	**FNB**
No. of patients	215	88	61
Age, mean (SD), y	73.50 (8.1)	73.23 (7)	70.97 (7.8)
Gender, No. (%),			
Male	134 (62.3)	51 (58)	43 (70.5)
Female	81 (37.7)	37 (42)	18 (29.5)
Age at PD onset, mean (SD), y	65.05 (9.8)	62.69 (10.5)	61.3 (10.3)
UPDRS Total score on state, mean (SD)	56.9 (24.6)	58.02 (27.7)	62.8 (29.1)
I	7.7 (6.4)	7.33 (6.5)	8.8 (6.7)
II	15.3 (7.9)	17.7 (8.9)	15.6 (9.3)
III	31.8 (13.4)	30.3 (14)	36 (15.7)
IV	2 (2.93)	2.7 (3.7)	2.1 (2.8)
Modified H&Y stage, mean (SD)	2.62 (0.8)	3.09 (0.9)	2.5 (0.9)
Disease Duration, mean (SD), y	8.02 (5.9)	10.22 (7.1)	9.5 (7.6)
Dominant Phenotype, *n* (%)			
Bradykinetic/Rigid type	106 (49.3)	47 (53.4)	26 (42.6)
Tremor type	33 (15.4)	20 (22.7)	18 (29.5)
Mixed Type	76 (35.3)	21 (23.9)	17 (27.9)
L-dopa equivalent daily dose, mg, mean (SD)	632.27 (349.54)	666.43 (339.76)	581.13 (330.23)

*SD, standard deviation; PD, Parkinson's Disease; PA, Postural Abnormalities including forward trunk bending (FTB), lateral trunk bending (LTB), and forward neck bending (FNB); y, years; H&Y, Hoehn and Yahr; UPDRS, Unified Parkinson's Disease Rating Scale*.

### Correlation of Clinical and Demographical Variables With Limitation in ADLs, Motor Impairment, Falls, and Pain

In the measures taken with the WG, the univariate logistic regression model yielded significant associations between upper/lower FTB, LTB, and FNB and many of the investigated clinical and demographic features (Tables 2–5). After adjusting for all variables in the model, multivariate logistic regression analysis confirmed the following associations.

*Upper FTB:* modified H&Y stage (OR 4.34, 95% CI 2.47–7.61) and disease duration (OR 1.08, 95% CI 1–1.17) were associated with limitations in ADLs. The modified H&Y stage was also associated with motor impairment (OR 2.43, 95% CI 1.53–3.85), back pain (OR 1.63, 95% CI 1.06–2.49), and falls (OR 2.06, 95% CI 1.27–3.32) (Table 2).
*Lower FTB:* modified H&Y stage was associated with limitations in ADLs (OR 6.54, 95% CI 1.79–23.78) and motor impairment (OR 6.03, 95% CI 1.67–21.67) (Table 3).
*LTB:* modified H&Y stage (OR 4.26, 95% CI 2.07–8.77) was associated with limitations in ADLs. Sex (OR 0.35, 95% CI 0.12–0.99), modified H&Y stage (OR 2.75, 95% CI 1.33–5.58), and degree of trunk bending (OR 1.12, 95% CI 1.03–1.22) were associated with motor impairment. Sex (OR 0.33, 95% CI 0.13–0.87) and modified H&Y stage (OR 1.86, 95% CI 1.01–3.41) were associated with back pain (Table 4).
*FNB:* modified H&Y stage (OR 3.37, 95% CI 1.33–8.53) was associated with motor impairment (Table 5).

**Table 2:** Clinical and demographic variables associated with upper FTB, as measured with a wall goniometer.

**Dependent variable**	**Independent variables**	Total^*^ sample	**Unadjusted**			**Adjusted**		
			**OR**	**95% CI**	***P*-value**	**OR**	**95% CI**	***P*-value**
Limitations in ADLs								
	Number of patients	188						
	Sex, males vs. females^∧^		0.99	0.5–.84	0.98	1.76	0.8–.82	0.15
	Modified H&Y stage		5.01	2.99–8.38	**<0.0005**	4.34	2.47–7.61	**<0.0005**
	Disease duration, y		1.15	1.08–1.23	**<0.0005**	1.08	1.00–1.17	**0.049**
	Degrees		1.06	1.02–1.09	**0.001**	1.02	0.98–1.06	0.38
Motor impairment								
	Number of patients	188						
	Sex, males vs. females^∧^		1.14	0.62–2.10	0.67	1.56	0.80–3.02	0.19
	Modified H&Y stage		2.13	1.44–3.15	**<0.0005**	2.43	1.53–3.85	**<0.0005**
	Disease duration, y		1.02	0.97–1.07	0.46	0.96	0.91–1.03	0.27
	Degrees		1.02	0.99- 1.06	0.13	1.01	0.97–1.04	0.64
Back pain								
	Number of patients	188						
	Sex, males vs. females^∧^		0.67	0.36–1.24	0.20	0.78	0.41–1.49	0.47
	Modified H&Y stage		1.81	1.24–2.64	**0.002**	1.63	1.06–2.49	**0.026**
	Disease duration, y		1.05	0.99–1.11	0.06	1.02	0.96–1.08	0.49
	Degrees		1.02	0.99–1.06	0.14	1.00	0.97–1.04	0.73
Falls								
	Number of patients	188						
	Sex, males vs. females^∧^		1.16	0.57–2.34	0.68	1.43	0.68–2.99	0.34
	Modified H&Y stage		1.77	1.17–2.67	**0.006**	2.06	1.27–3.32	**0.003**
	Disease duration, y		1.02	0.96–1.07	0.58	0.98	0.92–1.05	0.61
	Degrees		1	0.96–1.04	0.97	0.98	0.94–1.02	0.41

CI, confidence interval; H&Y, Hoehn and Yahr; OR, Odds Ratio; PAD, Parkinson disease; ∧, denotes the reference category; FTB, forward trunk bending;

* *The total sample included the isolated upper FTB (n = 175) and combined forms (upper + lower FTB, n = 13); significant associations in bold at p < 0.05*.

**Table 3:** Clinical and demographic variables associated with lower FTB, as measured with a wall goniometer.

**Dependent variable**	**Independent variables**	Total sample^*^	**Unadjusted**			**Adjusted**		
			**OR**	**95% CI**	***P*-value**	**OR**	**95% CI**	***P*-value**
Limitations in ADLs								
	Number of patients	40						
	Sex, males vs. females^∧^		0.60	0.17–2.16	0.43	0.77	0.17–3.56	0.74
	Modified H&Y stage		5.32	1.67–16.93	**0.005**	6.54	1.79–23.78	**0.004**
	Disease duration, y		0.97	0.88–1.07	0.57	0.92	0.81–1.05	0.21
	Degrees		1.01	0.95–1.07	0.71	1	0.94–1.07	0.94
Motor impairment								
	Number of patients	40						
	Sex, males vs. females^∧^		0.86	0.25–3.05	0.82	1.61	0.33–7.77	0.55
	Modified H&Y stage		4.64	1.57–13.74	**0.006**	6.03	1.67–21.67	**0.006**
	Disease duration, y		0.98	0.89–1.08	0.78	0.96	0.85–1.09	0.57
	Degrees		1.05	0.98–1.11	0.17	1.06	0.97–1.15	0.16
Back pain								
	Number of patients	40						
	Sex, males vs. females^∧^		2.09	0.52–8.46	0.30	1.65	0.36–7.64	0.52
	Modified H&Y stage		0.62	0.26–1.45	0.27	0.66	0.26–1.67	0.38
	Disease duration, y		0.98	0.89–1.08	0.70	0.95	0.85–1.06	0.36
	Degrees		0.94	0.88–1	0.06	0.93	0.87–1	0.06
Falls								
	Number of patients	40						
	Sex, males vs. females^∧^		2.22	0.62–7.98	0.22	3.74	0.85–16.51	0.08
	Modified H&Y stage		1.70	0.76–3.81	0.19	2.15	0.84–5.46	0.11
	Disease duration, y		0.96	0.88–1.06	0.48	0.97	0.87–1.08	0.59
	Degrees		1.04	0.98–1.11	0.18	1.05	0.98–1.13	0.17

CI, confidence interval; H&Y, Hoehn and Yahr; OR, Odds Ratio; PAD, Parkinson disease; ∧, denote the reference category; FTB, forward trunk bending;

* *The total sample included the isolated lower FTB (n = 27) and combined forms (upper + lower FTB, n = 13); significant associations in bold at p < 0.05*.

**Table 4:** Clinical and demographic variables associated with LTB, as measured with a wall goniometer.

**Dependent variable**	**Independent variables**	**Total sample**	**Unadjusted**			**Adjusted**		
			**OR**	**95% CI**	***P*-value**	**OR**	**95% CI**	***P*-value**
Limitations in ADLs								
	Number of patients	88						
	Sex, males vs. females^∧^		1.08	0.46–2.56	0.85	0.72	0.23–2.23	0.57
	Modified H&Y stage		4.66	2.46–8.82	**<0.0005**	4.26	2.07–8.77	**<0.0005**
	Disease duration, y		1.17	1.07–1.28	**0.001**	1.09	0.98–1.22	0.08
	Degrees		1.02	0.96–1.08	0.46	1.08	0.99–1.17	0.07
Motor impairment								
	Number of patients	88						
	Sex, males vs. females^∧^		0.54	0.22–1.29	0.16	0.35	0.12–0.99	**0.049**
	Modified H&Y stage		2.14	1.24–3.70	**0.006**	2.75	1.33–5.58	**0.005**
	Disease duration, y		1.06	0.99–1.13	0.07	1.02	0.95–1.10	0.56
	Degrees		1.06	0.99–1.13	0.06	1.12	1.03–1.22	**0.007**
Back pain								
	Number of patients	88						
	Sex, males vs. females^∧^		0.41	0.16–0.99	**0.049**	0.33	0.13–0.87	**0.024**
	Modified H&Y stage		1.53	0.94–2.94	0.08	1.86	1.01–3.41	**0.046**
	Disease duration, y		1.02	0.96–1.08	0.56	0.98	0.92–1.06	0.74
	Degrees		1.03	0.97–1.09	0.38	1.05	0.98–1.13	0.15
Falls								
	Number of patients	88						
	Sex, males vs. females^∧^		1.56	0.60–4.02	0.36	1.45	0.54–3.92	0.46
	Modified H&Y stage		1.61	0.93–2.79	0.09	1.82	0.96–3.44	0.07
	Disease duration, y		1.02	0.95–1.08	0.59	0.99	0.92–1.07	0.84
	Degrees		1.05	0.98–1.11	0.13	1.06	0.99–1.13	0.08

CI, confidence interval; H&Y, Hoehn and Yahr; OR, Odds Ratio; LTB, Lateral Trunk Bending;

∧ *denotes the reference category; significant associations in bold at p < 0.05*.

**Table 5:** Clinical and demographic variables associated with FNB, as measured with a wall goniometer.

**Dependent variable**	**Independent variables**	**Total sample**	**Unadjusted**			**Adjusted**		
			**OR**	**95% CI**	***P*-value**	**OR**	**95% CI**	***P-*value**
Limitations in ADLs								
	Number of patients	61						
	Sex, males vs. females^∧^		0.93	0.30–2.88	0.90	1.27	0.26–6.18	0.77
	Modified H&Y stage		3.98	1.92–8.23	**<0.0005**	2.32	0.97–5.50	0.06
	Disease duration, y		1.21	1.08–1.35	**0.001**	1.12	0.99–1.27	0.07
	Degrees		1.03	1–1.06	**0.027**	1.02	0.98–1.05	0.32
Motor impairment								
	Number of patients	61						
	Sex, males vs. females^∧^		0.73	0.24–2.25	0.58	0.93	0.24–3.54	0.91
	Modified H&Y stage		2.60	1.33–5.10	**0.005**	3.37	1.33–8.53	**0.010**
	Disease duration, y		1.06	0.98–1.14	0.16	0.96	0.86–1.07	0.50
	Degrees		1	0.98–1.03	0.85	0.99	0.96–1.02	0.57
Back pain								
	Number of patients	61						
	Sex, males vs. females^∧^		1.87	0.56–6.19	0.30	1.76	0.46–6.73	0.41
	Modified H&Y stage		1.29	0.74–2.25	0.36	2.16	0.93–5.02	0.07
	Disease duration, y		0.97	0.90–1.04	0.42	0.91	0.81–1.01	0.08
	Degrees		1.01	0.98–1.04	0.33	1.01	0.98–1.04	0.57
Falls								
	Number of patients	61						
	Sex, males vs. females^∧^		2.68	0.67–10.74	0.16	4.69	0.98–22.35	0.05
	Modified H&Y stage		1.04	0.57–1.86	0.90	0.85	0.35–2.07	0.72
	Disease duration, y		1.03	0.96–1.10	0.41	1.07	0.96–1.20	0.18
	Degrees		0.98	0.96–1.02	0.39	0.97	0.94–1.01	0.11

CI, confidence interval; H&Y, Hoehn and Yahr; OR, Odds Ratio; FNB, Forward Neck Bending;

∧ *denotes the reference category; y, years; significant associations in bold at p < 0.05*.

The SBM showed similar results in the adjusted and unadjusted ORs. No other statistically significant value was found for upper/lower FTB, LTB, and FNB measured with the WG and SBM (Tables 2–5 and Supplementary Tables 1–4).

### ROC Curve Analysis

Given the significant association in the multivariate logistic regression model, we performed ROC analysis to predict the LTB cut-off angle for best discriminating low vs. high disability. According to the clinical WG evaluation, the optimal cut-off value was 12.5°, with a sensitivity of 0.5 and a specificity of 0.8 (AUC 0.615; *p*: 0.071). According to the SBM, the optimal cut-off value was 10.5°, with a sensitivity of 0.5 and a specificity of 0.8 (AUC 0.626; *p*: 0.048).

## Discussion

With this study we wanted to analyze the influence of trunk bending severity on disability and determine the cut-off values of trunk bending that best discriminated limitations in ADLs, motor impairment, falls, and back pain. The adjusted odds ratios showed a significant association between motor impairment and degree of LTB, indicating that an increase in lateral bending increases the probability of greater motor impairment. We found that the optimal cut-off to identify moderate to severe motor impairment was 10.5°, corresponding to the most common cut-off for the diagnosis of PS (3, 11). In contrast, for upper/lower FTB and FNB we found no association between limitation of ADLs, motor impairment, pain, and falls and the degree of bending, except for a more advanced stage of disease (H&Y stage), longer disease duration, and sex. Our data cannot explain the association between the degree of LTB and female gender with motor impairment. However, we hypothesize that trunk asymmetry related to LTB may reduce trunk mobility more than FTB, leading to a greater impairment in motor functions, and intrinsic gender differences of the anatomy of the spine (24) may be a predisposing factor, along with subclinical osteoporosis for which the frequency in women is more than double that in men (25).

PD-associated postural abnormalities have attracted increasing attention from clinicians and researchers since they were recognized as a frequent and disabling complication of PD (1, 3–6, 26). Most studies on severe forward and lateral trunk flexion (i.e., CC, PS, and AC) to date have applied arbitrary and often diverse diagnostic criteria (2, 3). Despite the different criteria employed, greater disability is noted in patients with CC, PS, and AC than in those without severe postural abnormalities (1). Moreover, patients with lower CC seem to have more severe gait and postural control impairment than those with upper CC and without CC (26). Our data show that the severity of trunk bending cannot fully explain impairments. Patients with a more advanced PD stage and longer disease duration are at higher risk of having limitations in ADLs, motor impairment, back pain, and falls, independent of the degree of trunk bending. Our data are consistent with previous studies indicating that the association between postural abnormalities and such variables may be more closely related to the severity of the PD phenotype and disease progression than to the severity of trunk flexion itself (1, 5).

With regard to limitations in ADLs and perceived pain, a study uncorrected for possible confounders involving 145 subjects with PD and FTB did not yield convincing evidence for a threshold angle defining CC (27). Indeed, while showing a significant association between FTB ≥30° and limitations in ADLs and back pain, the authors remarked that less severe forward angles do not exclude CC (27). They found greater limitations in ADLs in patients with FTB ≥30°, which is in line with our univariate regression models reported for upper FTB. Nevertheless, here we demonstrate that this association did not survive multivariate logistic regression analysis, which indicated that sex, duration and stage of disease are relevant confounding factors.

Pain, one of the most frequent non-motor symptoms of PD, has been strongly associated with a worse quality of life (28–30). The relationship between PD and pain is complex and not fully elucidated; however, it has been demonstrated that PD patients with motor complications have a greater risk of developing pain (31), which seems to increase with worsening parkinsonian symptoms (28). Our findings suggest that the back pain in patients with trunk misalignment could be more closely related to the PD stage and duration than to the postural alterations.

Hence, the severity of trunk/neck bending alone does not explain limitation in ADLs, motor impairment, falls, and back pain in PD patients. Multiple factors possibly related to an aggressive PD phenotype account for disability. Nonetheless, less severe degrees of trunk bending—though not classified as CC, PS, or AC—should be monitored (i.e., quantitively by using apps) (32) and promptly treated (i.e., physiotherapy) (33) to prevent progression and worsening and to avoid permanent deformity. Physiotherapy should include active self-correction, trunk stabilization exercises (i.e., strengthening of compensatory paraspinal muscles) and functional tasks, along with pharmacological intervention such as botulinum toxin injection to reduce muscle hyperactivity (4, 14, 33, 34).

Some limitations should be taken into account when interpreting our results. First, the ROC analysis showed only a moderate accuracy of discrimination, as reflected by AUC values and the not very high values of sensitivity and specificity. Second, the necessity of a dichotomous outcome to run the ROC analysis required us to identify validated cut-offs for the patient-centered outcome measures. We could not find such cut-off values for Parkinson's disease Questionnaire 8, which would have extended the analysis to include quality of life. Third, the history of falls was based on patient-reported number of falls in the month before assessment, and no significant FTB, LTB, or FNB values were found for discriminating the history of falls. It is possible that analysis with more accurate recording of falls might have yielded significant results. Fourth, we did not evaluate as specific outcomes motor fluctuations, cognitive impairment, and freezing of gait which can be present in patients with PD and postural abnormalities (35) and associated with poor quality of life and falls. Finally, a prospective study design would help clarify the association and risk factors for disability in a cohort of patients with postural abnormalities.

These limitations notwithstanding, our findings indicate that advanced stage of disease and longer disease duration, but not the degree of trunk bending, may explain the greater disability of PD patients with postural abnormalities. Limitation in ADLs and presence history of falls and severe pain in FTB, LTB, and FNB may be more closely related to PD phenotype and progression than trunk flexion itself (1, 5). In contrast, moderate/severe motor impairment can be predicted by a degree of LTB > 10°, which is the cut-off typically used for the diagnosis of PS. This is an important finding because it justifies the widely used cut-off of 10° defined *a priori* by previous studies. Summarizing, all patients with PS should be referred to management by a multidisciplinary team at an early stage to improve their quality of life and slow the progress of motor decline (4).

## Ethics Statement

The studies involving human participants were reviewed and approved by Azienda Ospedaliera Universitaria Integrata Verona. The patients/participants provided their written informed consent to participate in this study. Written informed consent was obtained from the individual(s) for the publication of any potentially identifiable images or data included in this article.

## Author Contributions

CG, CA, and MT drafting and revising the manuscript, study concept and design, acquisition, analysis and interpretation of data, and study execution. MG, EZ, and RC drafting/revising the manuscript, study concept and design, interpretation of data, and study execution. MCap, EA, MGC, LB, MO, RT, GB, MCat, PM, SM, SG, LV, FS, MCas, CF-P, MZ, AF, and LL drafting/revising the manuscript, acquisition and interpretation of data, and study execution.

### Conflict of Interest

The authors declare that the research was conducted in the absence of any commercial or financial relationships that could be construed as a potential conflict of interest.
