# Supplementary material for: Does the Degree of Trunk Bending Predict Patient Disability, Motor Impairment, Falls, and Back Pain in Parkinson's Disease?
Source: Front Neurol. 2020 Mar 31;11:207. doi: 10.3389/fneur.2020.00207 (PMC7136533; doi:10.3389/fneur.2020.00207)
Supplement: Supplementary file 1 [file Table_1.docx]

| Supplementary Table 1. Clinical and demographic variables associated with upper FTB, as measured with software. | | | | | | | |
| --- | --- | --- | --- | --- | --- | --- | --- |
| Dependent variable Independent variables | Total sample* | Unadjusted | | | Adjusted | | |
|  |  | OR | 95% CI | P Value | OR | 95% CI | P Value |
| Limitations in ADLs |  |  |  |  |  |  |  |
| Number of patients | 188 |  |  |  |  |  |  |
| Sex, males vs. females^ |  | 0.99 | 0.53-1.84 | 0.98 | 1.73 | 0.80-3.73 | 0.16 |
| Modified H&Y stage |  | 5.01 | 2.99-8.39 | **<0.0005** | 4.43 | 2.53-7.74 | **<0.0005** |
| Disease duration, y |  | 1.15 | 1.08-1.23 | **<0.0005** | 1.08 | 1-1.17 | **0.04** |
| Degrees |  | 1.05 | 1.01-1.08 | **0.01** | 1.01 | 0.97-1.06 | 0.49 |
| Motor impairment |  |  |  |  |  |  |  |
| Number of patients | 188 |  |  |  |  |  |  |
| Sex, males vs. females ^ |  | 1.14 | 0.62-2.10 | 0.67 | 1.55 | 0.80-2.99 | 0.19 |
| Modified H&Y stage |  | 2.13 | 1.44-3.15 | **<0.0005** | 2.49 | 1.57-3.94 | **<0.0005** |
| Disease duration, y |  | 1.02 | 0.97-1.07 | 0.46 | 0.97 | 0.91-1.03 | 0.31 |
| Degrees |  | 1.01 | 0.98-1.05 | 0.43 | 0.99 | 0.96-1.03 | 0.92 |
| Back pain |  |  |  |  |  |  |  |
| Number of patients | 188 |  |  |  |  |  |  |
| Sex, males vs. females ^ |  | 0.67 | 0.36-1.24 | 0.20 | 0.79 | 0.42-1.49 | 0.47 |
| Modified H&Y stage |  | 1.81 | 1.24-2.64 | **0.002** | 1.70 | 1.11-2.60 | **0.014** |
| Disease duration, y |  | 1.05 | 0.99-1.11 | 0.06 | 1.02 | 0.96-1.08 | 0.42 |
| Degrees |  | 1.00 | 0.97-1.04 | 0.83 | 0.89 | 0.95-1.02 | 0.55 |
| Falls |  |  |  |  |  |  |  |
| Number of patients | 188 |  |  |  |  |  |  |
| Sex, males vs. females ^ |  | 1.16 | 0.57-2.34 | 0.68 | 1.48 | 0.70-3.11 | 0.30 |
| Modified H&Y stage |  | 1.77 | 1.17-2.67 | **0.006** | 2.11 | 1.31-3.41 | **0.002** |
| Disease duration, y |  | 1.02 | 0.96-1.07 | 0.58 | 0.98 | 0.92-1.05 | 0.62 |
| Degrees |  | 0.98 | 0.95-1.02 | 0.44 | 0.97 | 0.93-1.01 | 0.14 |
| Abbreviations: CI = confidence interval; H&Y = Hoehn and Yahr; OR = Odds Ratio; y, years; FTB, Forward Trunk Bending; ^= denotes the reference category; *the total sample included the isolated upper FTB (n=175) and combined forms (upper + lower FTB, n=13); Significant associations in bold at p <0.05. | | | | | | | |

| Supplementary Table 2. Clinical and demographic variables associated with lower FTB, as measured with software. | | | | | | | |
| --- | --- | --- | --- | --- | --- | --- | --- |
| Dependent variable Independent variables | Total sample | Unadjusted | | | Adjusted | | |
|  |  | OR | 95% CI | P Value | OR | 95% CI | P Value |
| Limitations in ADLs |  |  |  |  |  |  |  |
| Number of patients | 40 |  |  |  |  |  |  |
| Sex, males vs. females ^ |  | 0.60 | 0.17-2.16 | 0.43 | 0.77 | 0.16-3.66 | 0.75 |
| Modified H&Y stage |  | 5.32 | 1.67-16.93 | **0.005** | 6.53 | 1.79-23.75 | **0.004** |
| Disease duration. y |  | 0.97 | 0.88-1.07 | 0.57 | 0.92 | 0.81-1.05 | 0.22 |
| Degrees |  | 1.02 | 0.96-1.09 | 0.48 | 1 | 0.92-1.09 | 0.95 |
| Motor impairment |  |  |  |  |  |  |  |
| Number of patients | 40 |  |  |  |  |  |  |
| Sex, males vs. females ^ |  | 0.86 | 0.25-3.05 | 0.82 | 1.89 | 0.36-9.83 | 0.45 |
| Modified H&Y stage |  | 4.64 | 1.57-13.74 | **0.006** | 5.79 | 1.60-20.92 | **0.007** |
| Disease duration, y |  | 0.98 | 0.90-1.08 | 0.78 | 0.97 | 0.86-1.11 | 0.71 |
| Degrees |  | 1.07 | 0.99-1.15 | 0.08 | 1.08 | 0.98-1.18 | 0.11 |
| Back pain |  |  |  |  |  |  |  |
| Number of patients | 40 |  |  |  |  |  |  |
| Sex, males vs. females ^ |  | 2.09 | 0.52-8.46 | 0.30 | 1.49 | 0.33-6.81 | 0.61 |
| Modified H&Y stage |  | 0.62 | 0.26-1.45 | 0.27 | 0.69 | 0.28-1.73 | 0.43 |
| Disease duration, y |  | 0.98 | 0.89-1.08 | 0.70 | 0.94 | 0.24-1.06 | 0.33 |
| Degrees |  | 0.94 | 0.87-1.01 | 0.07 | 0.92 | 0.85-1.01 | 0.07 |
| Falls |  |  |  |  |  |  |  |
| Number of patients | 40 |  |  |  |  |  |  |
| Sex, males vs. females ^ |  | 2.22 | 0.62-7.98 | 0.22 | 3.59 | 0.82-15.75 | 0.09 |
| Modified H&Y stage |  | 1.70 | 0.76-3.81 | 0.19 | 2.06 | 0.82-5.14 | 0.12 |
| Disease duration, y |  | 0.96 | 0.88-1.06 | 0.48 | 0.97 | 0.87-1.08 | 0.56 |
| Degrees |  | 1.03 | 0.96-1.10 | 0.37 | 1.04 | 0.96-1.12 | 0.36 |
| Abbreviations: CI = confidence interval; H&Y = Hoehn and Yahr; OR = Odds Ratio; y, years; FTB, Forward Trunk Bending; ^= denotes the reference category; *the total sample included the isolated upper FTB (n=27) and combined forms (upper + lower FTB, n=13); Significant associations in bold at p <0.05. | | | | | | | |

| Supplementary Table 3. Clinical and demographic variables associated with LTB, as measured with software. | | | | | | | |
| --- | --- | --- | --- | --- | --- | --- | --- |
| Dependent variable Independent variables | Total sample | Unadjusted | | | Adjusted | | |
|  |  | OR | 95% CI | P Value | OR | 95% CI | P Value |
| Limitations in ADLs |  |  |  |  |  |  |  |
| Number of patients | 88 |  |  |  |  |  |  |
| Sex, males vs. females ^ |  | 1.08 | 0.46-2.56 | 0.85 | 0.64 | 0.20-2.04 | 0.45 |
| Modified H&Y stage |  | 4.66 | 2.46-8.82 | **<0.0005** | 4.38 | 2.13-9.02 | **<0.0005** |
| Disease duration, y |  | 1.17 | 1.07-1.28 | **0.001** | 1.09 | 0.98-1.22 | 0.10 |
| Degrees |  | 1.04 | 0.97-1.11 | 0.22 | 1.09 | 1-1.18 | **0.04** |
| Motor impairment |  |  |  |  |  |  |  |
| Number of patients | 88 |  |  |  |  |  |  |
| Sex, males vs. females ^ |  | 0.54 | 0.22-1.29 | 0.16 | 0.32 | 0.11-0.92 | **0.034** |
| Modified H&Y stage |  | 2.14 | 1.24-3.70 | **0.006** | 2.66 | 1.33-5.33 | **0.006** |
| Disease duration, y |  | 1.06 | 0.99-1.13 | 0.07 | 1.01 | 0.94-1.09 | 0.71 |
| Degrees |  | 1.06 | 0.99-1.12 | 0.06 | 1.11 | 1.03-1.19 | **0.008** |
| Back pain |  |  |  |  |  |  |  |
| Number of patients | 88 |  |  |  |  |  |  |
| Sex, males vs. females ^ |  | 0.41 | 0.16-0.94 | **0.049** | 0.30 | 0.11-0.81 | **0.017** |
| Modified H&Y stage |  | 1.53 | 0.94-2.49 | 0.08 | 1.89 | 1.03-3.47 | **0.040** |
| Disease duration, y |  | 1.02 | 0.95-1.08 | 0.56 | 0.98 | 0.91-1.06 | 0.66 |
| Degrees |  | 1.08 | 0.97-1.10 | 0.29 | 1.06 | 0.99-1.14 | 0.08 |
| Falls |  |  |  |  |  |  |  |
| Number of patients | 88 |  |  |  |  |  |  |
| Sex, males vs. females ^ |  | 1.56 | 0.60-4.02 | 0.36 | 1.34 | 0.49-3.63 | 0.56 |
| Modified H&Y stage |  | 1.61 | 0.93-2.79 | 0.09 | 1.82 | 0.96-3.46 | 0.06 |
| Disease duration, y |  | 1.02 | 0.95-1.08 | 0.59 | 0.98 | 0.92-1.06 | 0.73 |
| Degrees |  | 1.05 | 0.98-1.11 | 0.12 | 1.06 | 0.99-1.13 | 0.08 |
| Abbreviations: CI = confidence interval; H&Y = Hoehn and Yahr; OR = Odds Ratio; y, years; LTB, Lateral Trunk Bending; ^= denotes the reference category; Significant associations in bold at p <0.05. | | | | | | | |

| Supplementary Table 4. Clinical and demographic variables associated with FNB, as measured with software. | | | | | | | |
| --- | --- | --- | --- | --- | --- | --- | --- |
| Dependent variable Independent variables | Total sample | Unadjusted | | | Adjusted | | |
|  |  | OR | 95% CI | P Value | OR | 95% CI | P Value |
| Limitations in ADLs |  |  |  |  |  |  |  |
| Number of patients | 61 |  |  |  |  |  |  |
| Sex, males vs. females ^ |  | 0.93 | 0.30-2.88 | 0.90 | 1.29 | 0.27-6.26 | 0.75 |
| Modified H&Y stage |  | 3.98 | 1.93-8.23 | **<0.0005** | 2.36 | 0.99-5.57 | 0.05 |
| Disease duration, y |  | 1.21 | 1.08-1.35 | **0.001** | 1.12 | 0.98-1.26 | 0.09 |
| Degrees |  | 1.04 | 1.01-1.07 | **0.019** | 1.02 | 0.98-1.05 | 0.34 |
| Motor impairment |  |  |  |  |  |  |  |
| Number of patients | 61 |  |  |  |  |  |  |
| Sex, males vs. females ^ |  | 0.73 | 0.24-2.25 | 0.58 | 0.91 | 0.24-3.46 | 0.89 |
| Modified H&Y stage |  | 2.60 | 1.33-5.10 | **0.005** | 3.36 | 1.33-8.52 | **0.011** |
| Disease duration, y |  | 1.06 | 0.98-1.14 | 0.16 | 0.96 | 0.86-1.07 | 0.52 |
| Degrees |  | 1 | 0.98-1.03 | 0.81 | 0.99 | 0.96-1.02 | 0.59 |
| Back pain |  |  |  |  |  |  |  |
| Number of patients | 61 |  |  |  |  |  |  |
| Sex, males vs. females ^ |  | 1.87 | 0.56-6.19 | 0.30 | 1.66 | 0.43-6.38 | 0.46 |
| Modified H&Y stage |  | 1.29 | 0.74-2.25 | 0.36 | 2.14 | 0.92-4.96 | 0.08 |
| Disease duration, y |  | 0.97 | 0.90-1.04 | 0.42 | 0.90 | 0.80-1.01 | 0.07 |
| Degrees |  | 1.01 | 0.99-1.04 | 0.26 | 1.01 | 0.98-1.04 | 0.38 |
| Falls |  |  |  |  |  |  |  |
| Number of patients | 61 |  |  |  |  |  |  |
| Sex, males vs. females ^ |  | 2.68 | 0.67-10.74 | 0.16 | 4.36 | 0.93-20.45 | 0.06 |
| Modified H&Y stage |  | 1.04 | 0.57-1.86 | 0.90 | 0.85 | 0.35-2.03 | 0.71 |
| Disease duration, y |  | 1.03 | 0.96-1.10 | 0.41 | 1.07 | 0.97-1.19 | 0.17 |
| Degrees |  | 0.99 | 0.96-1.02 | 0.54 | 0.97 | 0.94-1.01 | 0.17 |
| Abbreviations: CI = confidence interval; H&Y = Hoehn and Yahr; OR = Odds Ratio; y, years; FNB, Forward Neck Bending; ^= denotes the reference category; Significant associations in bold at p <0.05. | | | | | | | |
